# Lesson from 610 liver resections of hepatocellular carcinoma in a single center over 10 years

**DOI:** 10.1186/1477-7819-12-192

**Published:** 2014-06-24

**Authors:** Dai Hoon Han, Gi Hong Choi, Jun Yong Park, Sang Hoon Ahn, Kyung Sik Kim, Jin Sub Choi, Kwang-Hyub Han

**Affiliations:** 1 Department of Surgery, Yonsei University College of Medicine, 250 Seongsanno, Seodaemoon-gu, Seoul 120-752, Korea; 2 Department of Internal Medicine, Yonsei University College of Medicine, Seoul, Korea; 3 Yonsei Liver Cancer Special Clinic, Yonsei University College of Medicine, Seoul, Korea

**Keywords:** Hepatocellular carcinoma, Hepatectomy, Prognosis, Survival

## Abstract

**Background:**

Recent advances in surgical techniques and perioperative management have led to improved surgical outcomes, especially perioperative outcomes. The aim of this study was to review our experience with hepatic resection for hepatocellular carcinoma (HCC) over a ten-year period to determine how to improve long-term surgical outcomes.

**Methods:**

From January 1996 to December 2007, 610 patients underwent curative resection for HCC at Yonsei University Health System, Seoul, Korea. Prognostic factors for disease-free and overall survival were identified, and surgical outcomes were compared between two time periods: before 2003 and after 2003.

**Results:**

The 1-, 3-, and 5-year overall survival rates were 90.1%, 74.9% and 64.4%, respectively. The patients after 2003 tended to have improved overall survival. The survival rate after recurrence in patients with tumors > 3 cm was significantly greater after 2003. (*P* = 0.044).

**Conclusions:**

The improved survival rates after 2003 may be explained by better selection of surgical candidates, a reduced perioperative transfusion rate due to improved surgical techniques, and active multimodal treatment for recurrent HCC.

## Background

The first successful liver resection was performed by Dr. Langenbuch in 1886 [1], but over the next 100 years, the surgical outcomes of liver resection improved only gradually. Until 1980, the hospital mortality rate could reach 33% after major hepatic resection, and operative death could exceed 15% [2].

However, recent advances in surgical techniques and perioperative management have led to improved surgical outcomes, especially perioperative outcomes. Some studies have reported that operative mortality and overall and disease-free survival rates have improved over the past two decades [3].

The purpose of this study was to review our experience with hepatic resection for hepatocellular carcinoma (HCC) over a ten-year period to determine how to improve long-term surgical outcomes by analyzing the impact of prognostic factors and active treatment for recurrent-HCC patients in two different time periods.

## Methods

### Patients

**Figure 1:**
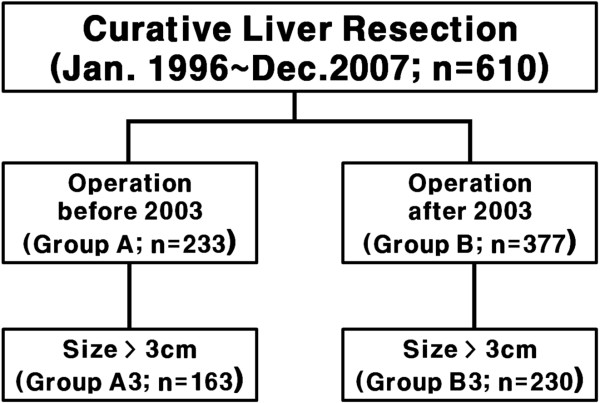
**Classification of patients for the study.** Patients were first divided into two groups according to the period of liver resection (group A: before 2003; group B: after 2003). Patients were then divided into two groups according to tumor size (group A3: larger than 3 cm; group B: smaller than 3 cm).

From January 1996 to December 2007, a total of 610 consecutive patients underwent curative liver resection for HCC at Yonsei University College of Medicine, Seoul, Korea. Curative resection was defined as complete removal of the tumor with a clear microscopic margin. All patients underwent pre-operative liver biochemistry tests and Child-Pugh grading. Surgical treatment was performed primarily in patients classified as Child-Pugh A; of the 610 patients, only 6 were Child-Pugh B (Table 1). The extent of resection was determined according to liver function such as the result of indocyanine green retention rate at 15 minutes (ICG R15), Child-Pugh classification, Clinical portal hypertension which might be represented with splenomegaly, thrombocytopenia and esophageal varix as well as gross findings of the liver during laparotomy. If ICG R15 was less than 10%, more than a right lobectomy was considered to be acceptable [4]. At our hospital, a tumor was considered resectable if there was no extrahepatic metastasis on preoperative imaging studies, no evidence of tumor thrombosis in major vessels such as the main portal vein or inferior vena cava, and if adequate tumor-free margins and sufficient remnant liver volumes were secured. Perioperative mortality was defined as death in the hospital after hepatectomy during the first admission for liver resection. Any complication requiring medication or an interventional procedure was considered perioperative morbidity.In 2003, our hospital established a Liver Cancer Special Clinic where hepatic surgeons, hepatologists, diagnostic radiologists, interventional radiologists, and radiation oncologists would meet once a week to discuss how to treat patients newly diagnosed with HCC and those with recurrent HCC. Since we expected that these multilateral diagnoses and treatment services would provide more effective treatment for HCC patients, we categorized patients in the current study according to the period of liver resection: before 2003, group A (n = 233), and after 2003, group B (n = 377). In a preliminary study, we also found that the overall survival of patients with tumors larger than 3 cm was greater after 2003 than before 2003. Thus, we also dichotomized patients with a tumor size above 3 cm according to the period of liver resection: before 2003, group A3 (n = 163), and after 2003, group B3 (n = 230, Figure 1).

**Table 1:** Characteristics of the 610 patients who received curative resection for hepatocellular carcinoma

Age (range)	53.36 ± 10.09 (22 to 81)
Gender (male/female)	480 (78.7%)/130 (21.3%)
Etiology	
HBV	476 (78.0%)
HCV	26 (4.3%)
both	8 (1.3%)
alcohol	12 (2.1%)
idiopathic	87 (14.3%)
Child-Pugh classification	
A/B	604 (99.0%)/6 (1.0%)
Cirrhosis	318 (52.1%)
Serum albumin (g/dL)	4.07 ± 0.51 (2.20 to 3.40)
AST (IU/L)	41.93 ± 28.80 (10 to 321)
ALT (IU/L)	42.30 ± 37.16 (3 to 425)
Operation	
minor/major	319 (52.3%)/291 (47.7%)
Perioperative bleeding	
≤ 1,000 cc/>1,000 cc	426 (69.8%)/184 (30.2%)
Perioperative transfusion	267 (43.8%)
Perioperative complication	169 (27.7%)
Perioperative mortality	12 (1.9%)
Resection margin	
≤ 1 cm/>1 cm	235 (38.5%)/356 (58.4%)
Tumor size (cm)	4.64 ± 2.81 (0.2 to 18.0)
Histologic differentiation	
Edmondson-Steiner grade I to II	358 (58.7%)
Edmondson-Steiner grade III to IV	156 (25.6%)
AFP (IU/mL)	2,541.38 ± 8,750.95 (0.46 to 83000.00)
Multiple tumors	122 (20.0%)
Gross vascular invasion	46 (7.5%)
Microscopic vascular invasion	304 (49.8%)

AFP, alpha-fetoprotein; ALT, alanine aminotransferase; AST, aspartate aminotransferase; HBV, hepatitis B virus; HCV, hepatitis C virus.

Each patient was screened postoperatively for tumor markers, such as alpha-fetoprotein (AFP) and protein-induced vitamin K absence or antagonist II (PIVKA-II), and underwent ultrasonography or a dynamic computed tomography scan one month after surgery and every three months thereafter. The median follow-up time after resection was 52 months (range: 0 to 152 months). If tumors recurred, appropriate treatment was initiated according to the number, size, and location of the tumors.

### Analysis of prognostic factors for disease-free and overall survival

Prognostic factors for disease-free and overall survival were investigated through 18 clinicopathologic variables, including patient-, surgical-, and tumor-related variables. Patient- related variables were age, sex, serum albumin, alanine aminotransferase (ALT), aspartate aminotransferase (AST), platelet count, and liver cirrhosis. Surgical variables were perioperative transfusion, amount of intraoperative bleeding, perioperative morbidity, surgical-free margin, and extent of resection. The extent of resection was determined according to Couinaud’s classification: a major resection was defined as a resection of three or more segments, and a minor resection, as a resection of two or fewer segments [5]. Tumor -related variables were tumor size, macroscopic vascular invasion, microscopic vascular invasion, histologic differentiation of the tumor by the Edmondson-Steiner grade, serum alpha-fetoprotein level, and multiplicity of tumors. Tumor size was classified into two groups: the patients with tumor less than or equal to 3 cm and those with tumor size greater than 3 cm. In cases of multiple tumors, the largest diameter of the main mass was considered to be the tumor size. Multiple tumors were defined as tumors with hepatic satellitosis, multifocal tumors, or intrahepatic metastases according to the seventh AJCC [6].

### Statistical analysis

Continuous data were analyzed with Student’s *t*-tests and presented as mean and standard deviation. Categorical variables were compared with χ^2^ tests or Fisher exact tests, as appropriate. Survival curves were obtained by the Kaplan-Meier method, and differences in survival between groups were compared with the log rank test. The 18 clinicopathologic variables were dichotomized and analyzed in relation to their effects on disease-free and overall survival after resection. Perioperative mortalities were included in the overall survival analysis but excluded from the disease-free survival analysis. After univariate analysis of the factors affecting survival, only the significant variables were used in the multivariable analysis with the Cox-proportional hazards model with the forward stepwise logistic regression model. Statistical analyses were performed with SPSS 18 for Windows (SPSS Inc, Chicago, IL, USA). A value of *P* < 0.05 was considered statistically significant.

## Results

### Clinicopathological features of all patients

Four hundred and eighty of 610 patients were men (78.7%). The most common underlying liver disease was hepatitis B viral infection (n = 476, 78.0%). The majority of patients had relatively good liver function: most were Child-Pugh class A with mean values of serum albumin, AST, and ALT in the normal range. More than half of patients had liver cirrhosis on histologic examination after operation (n = 318, 52.1%), and the mean tumor size was 4.64 cm (0.2 cm to 18.0 cm). One fifth of patients had multiple tumors (n = 122, 20.0%). About half of patients (n = 291, 47.7%) had undergone major liver resection. The amount of perioperative bleeding was below 1,000 cc in 436 patients (69.8%). The major cause of excessive perioperative bleeding was major liver resection (*P* = 0.014). There were 169 patients with perioperative complications (27.7%) and 12 with perioperative mortality (1.9%). Three hundred and fifty-six patients (58.4%) had a resection margin greater than 1 cm. On histopathologic examination, 358 patients had well- to moderate-differentiation (58.7%), 46 patients had gross vascular invasion (7.5%), and 304 patients had microscopic vascular invasion (49.8%) (Table 1).

### Prognostic factors for disease-free and overall survival of all patients

The 1-, 3-, and 5-year disease-free survival rates were 71.7%, 52.7%, and 45.8% (n = 598), respectively; the overall survival rates were 90.1%, 74.9%, and 64.4% (n = 610), respectively. Multivariable analysis revealed that AST (>50 IU/L), liver cirrhosis, perioperative transfusion, multiple tumors, microvascular invasion, and Edmondson-Steiner grade III to IV were independent adverse prognostic factors for disease-free survival; while serum albumin (≤3.5 g/dL), low platelet count (≤100,000 mm^3)^, perioperative transfusion, perioperative morbidity, tumor size larger than 3 cm, multiple tumors, macrovascular invasion, and Edmondson-Steiner grade III to IV were independent adverse prognostic factors for overall survival (Table 2). Thus, perioperative transfusion, liver cirrhosis, and Edmondson-Steiner grade III to IV were adverse prognostic factors for both disease-free and overall survival.

**Table 2:** Independent prognostic factors for disease-free and overall survival by multivariate analysis

**Variable**	**Coefficient**	**Standard error**	***P*-value**	**Relative risk (95% CI)**
Disease-free survival				
AST (>50 IU/L)	0.381	0.136	0.016	1.379 (1.062 to 1.789)
Liver cirrhosis	0.296	0.119	0.043	1.292 (1.009 to 1.654)
Perioperative transfusion	0.301	0.118	0.008	1.386 (1.090 to 1.763)
Multiple tumors	0.553	0.134	0.013	1.564 (1.097 to 2.230)
Microvascular invasion	0.382	0.128	< 0.001	1.604 (1.255 to 2.050)
Edmondson-Steiner grade III to IV	0.321	0.131	0.014	1.379 (1.066 to 1.784)
Overall survival				
Serum albumin (≤3.5 g/dL)	0.587	0.166	< 0.001	1.798 (1.298 to 2.490)
Platelet count (≤100,000/mm^3^)	0.442	0.167	0.008	1.557 (1.122 to 2.161)
Perioperative transfusion	0.477	0.140	0.001	1.610 (1.224 to 2.118)
Perioperative morbidity	0.353	0.143	0.013	1.423 (1.076 to 1.883)
Tumor size (>3 cm)	0.313	0.154	0.043	1.367 (1.010 to 1.850)
Multiple tumors	0.679	0.147	< 0.001	1.973 (1.478 to 2.633)
Macrovascular invasion	0.581	0.227	0.011	1.789 (1.145 to 2.793)
Edmondson-Steiner grade III to IV	0.419	0.147	0.004	1.521 (1.141 to 2.028)

AST, aspartate aminotransferase; CI, confidence interval.

### Disease-free and overall survival in relation to the period of hepatic resection

Neither disease-free nor overall survival differed significantly between groups (disease-free: *P* = 0.616; overall: *P* = 0.098). Nonetheless, there was a marked difference in overall survival between groups until 72 months; the 1-, 3-, and 5-year overall survival rates of the patients in group A were 87.1%, 69.5%, and 60.5%, respectively, whereas those of the patients in group B were 92.0%, 78.3%, and 66.4%, respectively (*P* = 0.098). Patients in group B were significantly older and had a greater incidence of liver cirrhosis, major resection, and surgical resection margin longer than 1 cm. Patients in group A had a greater incidence of low albumin, perioperative transfusion, minor resection, tumor size larger than 3 cm, multiple tumors, microvascular invasion, and Edmondson-Steiner grade III to IV.

### Overall survival after recurrence of HCC

Since overall survival tended to be greater in group B, the data for patients with recurrent HCC after liver resection were further evaluated. During the follow-up period, HCC recurred in 299 of 610 patients (49.2%). The 1-, 3-, and 5-year overall survival rates after recurrence were 74.6%, 50.7%, and 34.0%, respectively. Of the 299 patients with tumor recurrence, 282 underwent treatment for recurrence; the most commonly performed treatment was transarterial chemoembolization (TACE, 58.1%). More than 80% of deaths were due to tumor recurrence (Table 3). In addition, the 1-, 3-, and 5-year overall survival rates of patients with recurrent HCC were significantly lower before 2003 than after 2003 (*P* = 0.044). Specifically, the 1-, 3-, and 5-year overall survival rates after 2003 were 77.7%, 55.5%, and 39.9%, respectively, while the 1-, 3-, and 5-year overall survival rates before 2003 were 70.5%, 44.7%, and 28.5%, respectively.

**Table 3:** Recurrence of hepatocellular carcinoma and death after curative resection

**Recurrence**	
Intrahepatic recurrence	222 (76.6%)
Extrahepatic recurrence	68 (23.4%)
**Main treatment for recurrence**	
Transplantation	10 (3.4%)
Repeat resection	29 (9.8%)
Local ablation therapy	29 (9.8%)
TACE	172 (58.1%)
Chemotherapy	26 (4.3%)
Radiation therapy	16 (5.4%)
**Cause of death**	
Recurrent HCC	182 (80.9%)
Complication of liver cirrhosis without recurrence	25 (11.1%)
Unrelated (including perioperative death)	18 (8.0%)

HCC, hepatocellular carcinoma; TACE, transarterial chemoembolization.

### Overall survival of patients with tumors larger than 3 cm

**Figure 2:**
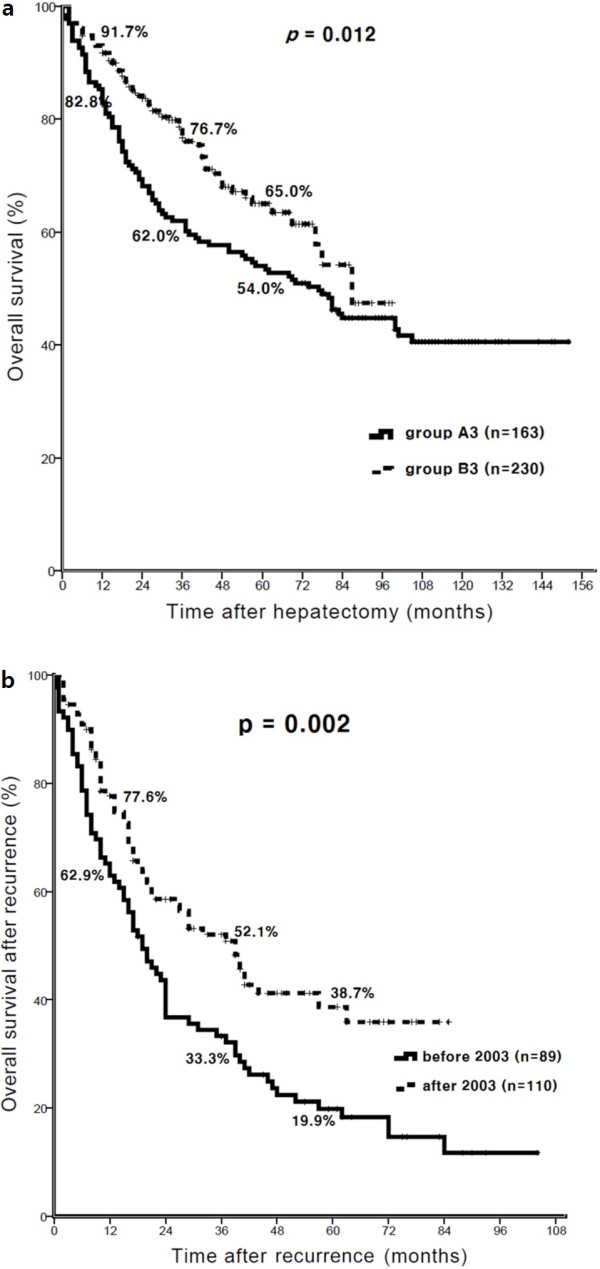
**Comparison of overall survival between groups A3 and B3. (a)** Overall survival of patients with tumors larger than 3 cm was significantly improved after 2003 (*P* = 0.012). **(b)** The overall survival after recurrence in patients with tumors larger than 3 cm was significantly improved after 2003 (*P* = 0.002).

In a preliminary study, we found no significant difference in disease-free survival after hepatectomy between patients with tumors larger than 3 cm and those with tumors smaller than 3 cm; however, overall survival was significantly lower for patients with tumors larger than 3 cm (*P* = 0.006). In the current study, there was no improvement in disease-free (*P* = 0.860) or overall survival (*P* = 0.224) in patients with tumors smaller than 3 cm after 2003. In contrast, the overall survival of patients with tumors larger than 3 cm was significantly improved after 2003 (*P* = 0.012, Figure 2a). Moreover, the overall survival after recurrence in the patients with tumors larger than 3 cm was significantly improved after 2003 (*P* = 0,002, Figure 2b). Patients in group A3 were more likely to have a preoperative serum albumin level lower than 3.5 g/dL (*P* < 0.001). Patients in group B3 were more likely to have had a major operation (*P* = 0.004). Perioperative transfusion (*P* < 0.001) and a safety resection margin shorter than 1 cm (*P* < 0.002) were more common in group A3. Multiple tumors (*P* = 0.008) and tumors with microscopic vascular invasion (*P* = 0.003) were more common in group A3 (Table 4). Male sex, serum albumin lower than 3.5 g/dL, liver cirrhosis, perioperative transfusion, intraoperative bleeding greater than 1,000 cc, perioperative complications, multiple tumors, and Edmondson-Steiner grade III to IV were independent adverse prognostic factors for overall survival of patients with tumors larger than 3 cm in multivariate analysis.

**Table 4:** Characteristics of patients with tumors larger than 3 cm

**Variables**	**Group A3 (n = 163)**	**Group B3 (n = 230)**	***P*-value**
Age (range)	51.90 ± 10.14	54.59 ± 9.69	0.008
Gender (male/female)	131/32 (80.4/19.6%)	177/53 (77.0/23.0%)	0.418
Etiology			0.085
HBV	125 (76.7%)	176 (76.5%)	
HCV	2 (1.2%)	12 (5.2%)	
both	1 (0.6%)	3 (1.3%)	
alcohol	7 (4.3%)	3 (1.3%)	
idiopathic	28 (17.2%)	36 (15.7%)	
Child-Pugh classification			0.653
A/B	160/3 (98.2/1.8%)	228/2 (99.1/0.9%)	
Cirrhosis	66 (40.5%)	114 (49.6%)	0.075
Serum albumin ≤ 3.5 g/dL	39 (24.1%)	25 (10.9%)	< 0.001
AST (IU/L) > 50	31 (19.1%)	56 (24.3%)	0.221
ALT (IU/L) > 50	38 (23.5%)	56 (24.3%)	0.839
Operation			0.004
minor/major operation	87/76 (53.4/46.6%)	89/141 (38.7/61.3%)	
Perioperative bleeding			0.131
≤ 1,000 cc/>1,000 cc	103/60 (63.2/36.8%)	162/68 (70.4/29.6%)	
Perioperative transfusion	109 (66.9%)	79 (34.3%)	< 0.001
Perioperative complication	51 (31.3%)	61 (26.5%)	0.302
Perioperative mortality	5 (3.1%)	5 (2.2%)	0.747
Resection margin			0.002
≤ 1 cm/>1 cm	79/72 (52.3/47.7%)	83/144 (36.6/63.4%)	
Histologic differentiation			0.073
Edmondson-Steiner grade III to IV	57 (35.0%)	57 (24.8%)	
AFP (IU/mL)	4,129.58 ± 11,213.53	3,086.58 ± 10,003.07	0.334
Multiple tumors	45 (27.6%)	38 (16.5%)	0.008
Gross vascular invasion	10 (6.1%)	22 (9.6%)	0.746
Microscopic vascular invasion	103 (63.2%)	111 (48.3%)	0.003

AFP, alpha-fetoprotein; ALT, alanine aminotransferase; AST, aspartate aminotransferase; HBV, hepatitis B virus; HCV, hepatitis C virus.

## Discussion

HCC is one of the most fatal malignant diseases. In Korea, HCC is the second most common cause of death from cancer even though it is the fifth most common newly diagnosed cancer [7]. Hepatic resection is considered to be the treatment of choice for HCC in patients with preserved liver function [8]. However, high perioperative morbidity and mortality have been major obstacles to overcome. Fortunately, recent advances in surgical techniques and perioperative management have led to improved surgical outcomes, especially perioperative outcomes [9-11]. Indeed, we observed improved surgical outcomes in the current study: the rate of perioperative morbidity was 27.7% and the rate of mortality, 1.9%, during the last 10 years. Thus, the major concern for researchers and clinicians has shifted from surgical technique to prognostic factors that predict successful outcomes. Various prognostic factors have been studied and are generally classified into three categories: patient, surgical, and tumor factors [12]. In this study, we investigated 18 prognostic factors, including patients factors (which are age, gender, serum albumin, ALT, AST, platelet count, and liver cirrhosis), surgical factors (which are perioperative transfusion, perioperative bleeding, perioperative morbidity, surgical resection margin, and extent of resection), and tumor factors (which are tumor size, number of tumors, macroscopic vascular invasion, microscopic vascular invasion, histologic grades, and serum AFP level). Multivariable analysis revealed that these three factors each affected the long-term surgical outcomes.

Liver cirrhosis and AST levels, which were independent prognostic factors for disease-free survival in the current study, reflect the condition of the liver parenchyma around the tumor. Poon *et al*. [13] analyzed risk factors in relation to recurrence time after liver resection for patients with HCC and found that rupture of tumor and vascular invasion were risk factors for early recurrence, whereas liver cirrhosis was a risk factor for late recurrence. Therefore, the serum AST level, which reflects the activity of hepatitis and cirrhosis, might be related to recurrence by multicentric carcinogenesis rather than intrahepatic metastasis. Thus, controlling hepatitis activity and the progression of liver cirrhosis may help prevent tumor recurrence after liver resection. Mazzafero *et al*. [14] reported that interferon may reduce late tumor recurrence (that is two years after liver resection) in patients with hepatitis C. Similarly, Lo *et al*. [15] reported that patients with predominantly hepatitis B-related HCC might receive a survival benefit from adjuvant interferon. However, the study at our institution found that survival after treatment in patients with late recurrence did not differ from that of patients without recurrence after liver resection [16]. These results indicate that early detection and active treatment for recurrent HCC after liver resection is effective. This explains why serum AST level and liver cirrhosis were independent prognostic factors for disease-free survival, but not for overall survival, after liver resection.

Perioperative transfusion was an independent prognostic factor for both disease-free and overall survival. A few previous studies have also reported that HCC often recurs after perioperative transfusion [17-19]. This relationship might result from the immune suppressive effects of transfusion, in which the natural killer cells and lymphokine-activated killer cells are suppressed by reduction of IL-2 secretion from T cells [20].

Liver function at operation is closely related to postoperative morbidity. Moreover, liver function is also required to cope with the aggressive treatment required for HCC recurrence. In the current study, preoperative serum albumin level and platelet count, which reflect liver function and severity of liver cirrhosis, were independent prognostic factors for overall survival after liver resection. The 1-, 3-, and 5-year overall survival rates of the 24 patients with serum albumin levels lower than 3.5 g/dL and platelet counts lower than 100,000 mm^3^ were 75.0%, 45.1%, and 40.6%, respectively. These rates were significantly lower than those for patients with serum albumin levels above 3.5 g/dL and platelet counts above 100,000 mm^3^ (90.7%, 76.1%, and 65.8%, respectively; *P* = 0.001). Thus, minimal resection to preserve the normal parenchyma of the liver or other treatment modalities such as percutaneous ablation therapy or transplantation should be considered for patients with deteriorated liver function [21].

Multiple tumors, microvascular invasion, and a higher Edmondson-Steiner grade were independent prognostic factors for both disease-free and overall survival. These variables represent the aggressiveness of the tumor. Large tumor size is a well-known risk factor for recurrence after liver resection [18]. According to Shimozawa *et al*. [22], survival and recurrence rates in HCC tumors with a diameter smaller than 3 cm were better than those for tumors with a diameter larger than 3 cm. Similarly, our results indicate that a tumor size larger than 3 cm was an independent adverse prognostic factor for overall survival. We also compared the overall survival after tumor recurrence for patients with tumors larger than 3 cm in relation to the period of operation. Overall survival after recurrence was significantly greater for patients who underwent liver resection after 2003 than those who underwent liver resection before 2003 (*P* = 0.012, Figure 2a). Low serum albumin level, liver cirrhosis, perioperative transfusion, perioperative bleeding, perioperative morbidity, multiple tumors, and poor histologic grade were independent adverse prognostic factors for patients with tumors larger than 3 cm. Perioperative transfusion and clear resection margin less than 1 cm were more prevalent before 2003 (Table 4). The serum albumin level was higher in patients who underwent liver resection after 2003, which might indicate improvement in patient selection for liver resection. Perioperative transfusion was performed less frequently after 2003, although major liver resection was performed more frequently. These data suggest that surgical techniques had become more refined after 2003. Moreover, after 2003, our hospital had more effective and appropriate treatment modalities for patients with HCC recurrence due to the opening of our Liver Cancer Special Clinic. Thus, proper selection of surgical patients, advanced surgical techniques, and active treatment for patients with recurred HCC may have led to improved survival after recurrence. However, overall survival after recurrence in patients with tumors less than 3 cm did not differ before or after 2003 (*P* = 0.105). Hence, surgical technique and active treatment for recurrence may have a greater effect on the long-term survival of patients with larger than 3 cm HCC comparing those with smaller than 3 cm tumors.

Even after curative treatment for HCC, recurrence of HCC is quite common, with a 5-year recurrence rate reaching 80 to 90% [23]. In the current study, the 5-year disease-free survival rate after liver resection was 45.9%, and the overall survival rate was 64.4%. The majority of deaths (182 of 225; 80.9%) occurred in patients with recurrent HCC. The most common type of recurrence was intrahepatic recurrence (n = 222, 76.6%, Table 3), which is consistent with the results of previous studies [18,24-29]. Since this suggests that most deaths were related to recurrence after curative resection, it appears that the prevention or treatment of recurrence may improve long-term surgical outcomes after liver resection. Recent studies have described different treatments for recurrence, such as liver transplantation, liver resection, ablative therapy, or transarterial chemoembolization [22,25-28]. These multimodal treatments for recurrence were also performed in our hospital, and more active and meticulous approaches were possible after the establishment of the Liver Cancer Special Clinic in 2003. The 5-year overall survival rates were about 20% greater than the 5-year disease-free survival rates in this study. Due to these efforts, the overall survival after recurrence of HCC was significantly greater after 2003.

Our study has some limitations. First, the data were collected retrospectively. Second, although we believe that more active and appropriate treatment for recurrence was provided after 2003 by the Liver Cancer Special Clinic, there was no unequivocal evidence for this possibility. Therefore, a randomized controlled trial that includes the use of a clinical grading system to assess the activity and appropriateness of treatment for recurrence is required.

## Conclusions

**Figure 3:**
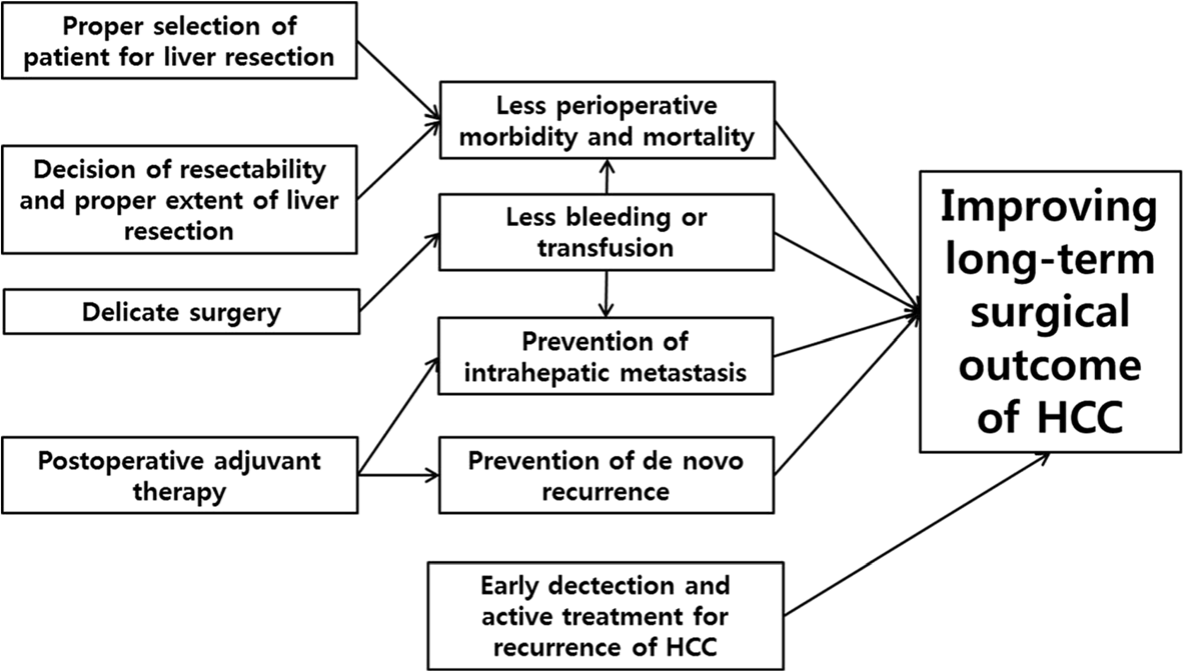
The practical strategy to improve long-term surgical outcomes of hepatocellular carcinoma.

On the basis of our ten-year experience of liver resection for HCC, we describe possible strategies to improve long-term surgical outcome for HCC in Figure 3. In brief, proper selection of surgical candidates, careful surgery to avoid perioperative bleeding or transfusion, early detection with active treatment for patients with recurrent HCC, and, possibly, postoperative adjuvant therapy for prevention of intrahepatic metastasis and *de novo* recurrence may be the key strategies to improve long-term survival after liver resection for HCC. Moreover, surgical treatment and active treatment for recurrence should be performed in patients with tumors larger than 3 cm.

## Abbreviations

AFP: alpha-fetoprotein; ALT: alanine aminotransferase; AST: aspartate aminotransferase; HCC: hepatocellular carcinoma; ICG R15: indocyanine green retention rate at 15 minutes; IL: interleukin; PIVKA-II: protein-induced vitamin K absence or antagonist II; TACE: transarterial chemoembolization.

## Competing interests

The authors declare that they have no competing interests.

## Authors’ contributions

DHH contributed mainly in the design, literature review and writing of the article. Collection and assembly of the data was performed by GHC and DHH. JSC provided the idea, planned, edited and approved the written work. GHC, JYP, SHA, KSK, JSC and KHH gave valuable advice and edited the discussion. Both JSC and GHC also provided administrative supports. All authors read and approved the manuscript.

## References

[B1] Hardy KJ Liver surgery: the past 2000 years Aust N Z J Surg 1990 60 10 811 817 10.1111/j.1445-2197.1990.tb07479.x 2206121

[B2] Nagao T Inoue S Mizuta T Saito H Kawano N Morioka Y One hundred hepatic resections. Indications and operative results Ann Surg 1985 202 1 42 49 10.1097/00000658-198507000-00006 2990359 PMC1250834

[B3] Fan ST Mau Lo C Poon RT Yeung C Leung Liu C Yuen WK Ming Lam C Ng KK Ching Chan S Continuous improvement of survival outcomes of resection of hepatocellular carcinoma: a 20-year experience Ann Surg 2011 253 4 745 758 10.1097/SLA.0b013e3182111195 21475015

[B4] Makuuchi MF Kosuge T Kosuge TF Takayama T Takayama TF Yamazaki S Yamazaki SF Kakazu T Kakazu TF Miyagawa S Miyagawa SF Kawasaki S Kawasaki S Surgery for small liver cancers Semin Surg Oncol 19938756–0437 (Print) 10.1002/ssu.2980090404 8210909

[B5] Couinaud C Liver anatomy: portal (and suprahepatic) or biliary segmentation Dig Surg 1999 16 6 459 467 10.1159/000018770 10805544

[B6] Edge SB Byrd DR Compton CC Fritz AG Greene FL Trotti A, editors: AJCC cancer staging manual (7th ed) 2010 New York: Springer

[B7] Jung KW Park S Won YJ Kong HJ Lee JY Park EC Lee JS Prediction of cancer incidence and mortality in Korea, 2011 Cancer Res Treat 2011 43 1 12 18 10.4143/crt.2011.43.1.12 21509158 PMC3072530

[B8] Lee SS Shin HS Kim HJ Lee SJ Lee HS Hyun KH Kim YH Kwon BW Han JH Choi H Kim BH Lee JH Kang HY Shin HD Song IH Analysis of prognostic factors and 5-year survival rate in patients with hepatocellular carcinoma: a single-center experience Korean J Hepatol 2012 18 1 48 55 10.3350/kjhep.2012.18.1.48 22511903 PMC3326996

[B9] Cescon M Vetrone G Grazi GL Ramacciato G Ercolani G Ravaioli M Del Gaudio M Pinna AD Trends in perioperative outcome after hepatic resection: analysis of 1500 consecutive unselected cases over 20 years Ann Surg 2009 249 6 995 1002 10.1097/SLA.0b013e3181a63c74 19474679

[B10] de Boer MT Molenaar IQ Porte RJ Impact of blood loss on outcome after liver resection Dig Surg 2007 24 4 259 264 10.1159/000103656 17657150

[B11] Yang T Zhang J Lu JH Yang GS Wu MC Yu WF Risk factors influencing postoperative outcomes of major hepatic resection of hepatocellular carcinoma for patients with underlying liver diseases World J Surg 2011 35 9 2073 2082 10.1007/s00268-011-1161-0 21656309

[B12] Qin L Tang Z The prognostic significance of clinical and pathological features in hepatocellular carcinoma World J Gastroenterol 2002 8 2 193 199 11925590 10.3748/wjg.v8.i2.193 PMC4658349

[B13] Poon RT Fan ST Ng IO Lo CM Liu CL Wong J Different risk factors and prognosis for early and late intrahepatic recurrence after resection of hepatocellular carcinoma Cancer 2000 89 3 500 507 10.1002/1097-0142(20000801)89:3<500::AID-CNCR4>3.0.CO;2-O 10931448

[B14] Mazzaferro V Romito R Schiavo M Mariani L Camerini T Bhoori S Capussotti L Calise F Pellicci R Belli G Tagger A Colombo M Bonino F Majno P Llovet JM Prevention of hepatocellular carcinoma recurrence with alpha-interferon after liver resection in HCV cirrhosis Hepatology 2006 44 6 1543 1554 10.1002/hep.21415 17133492

[B15] Lo CM Liu CL Chan SC Lam CM Poon RT Ng IO Fan ST Wong J A randomized, controlled trial of postoperative adjuvant interferon therapy after resection of hepatocellular carcinoma Ann Surg 2007 245 6 831 842 10.1097/01.sla.0000245829.00977.45 17522506 PMC1876947

[B16] Choi GH Kim DH Choi SB Kang CM Kim KS Choi JS Lee WJ Han KH Chon CY Kim BR The preoperative positivity for serum hepatitis B e antigen did not affect overall survival after curative resection of hepatitis B virus-related hepatocellular carcinoma J Gastroenterol Hepatol 2009 24 3 391 398 10.1111/j.1440-1746.2008.05637.x 19032452

[B17] Asahara T Katayama K Itamoto T Yano M Hino H Okamoto Y Nakahara H Dohi K Moriwaki K Yuge O Perioperative blood transfusion as a prognostic indicator in patients with hepatocellular carcinoma World J Surg 1999 23 7 676 680 10.1007/PL00012367 10390585

[B18] Tung-Ping Poon R Fan ST Wong J Risk factors, prevention, and management of postoperative recurrence after resection of hepatocellular carcinoma Ann Surg 2000 232 1 10 24 10.1097/00000658-200007000-00003 10862190 PMC1421103

[B19] Yamamoto J Kosuge T Takayama T Shimada K Yamasaki S Ozaki H Yamaguchi N Mizuno S Makuuchi M Perioperative blood transfusion promotes recurrence of hepatocellular carcinoma after hepatectomy Surgery 1994 115 3 303 309 8128355

[B20] Gascn P Zoumbos NC Young NS Immunologic abnormalities in patients receiving multiple blood transfusions Ann Intern Med 1984 100 2 173 177 10.7326/0003-4819-100-2-173 6229206

[B21] Dahiya D Wu TJ Lee CF Chan KM Lee WC Chen MF Minor versus major hepatic resection for small hepatocellular carcinoma (HCC) in cirrhotic patients: a 20-year experience Surgery 2010 147 5 676 685 10.1016/j.surg.2009.10.043 20004441

[B22] Shimozawa N Hanazaki K Longterm prognosis after hepatic resection for small hepatocellular carcinoma J Am Coll Surg 2004 198 3 356 365 10.1016/j.jamcollsurg.2003.10.017 14992736

[B23] Kudo M Adjuvant therapy after curative treatment for hepatocellular carcinoma Oncology 2011 81 s1 50 55 10.1159/000333259 22212936

[B24] Cha C Fong Y Jarnagin WR Blumgart LH DeMatteo RP Predictors and patterns of recurrence after resection of hepatocellular carcinoma J Am Coll Surg 2003 197 5 753 758 10.1016/j.jamcollsurg.2003.07.003 14585409

[B25] Lee PH Lin WJ Tsang YM Hu RH Sheu JC Lai MY Hsu HC May W Lee CS Clinical management of recurrent hepatocellular carcinoma Ann Surg 1995 222 5 670 676 10.1097/00000658-199511000-00010 7487215 PMC1234995

[B26] Poon RT Fan ST Lo CM Liu CL Wong J Intrahepatic recurrence after curative resection of hepatocellular carcinoma: long-term results of treatment and prognostic factors Ann Surg 1999 229 2 216 222 10.1097/00000658-199902000-00009 10024103 PMC1191634

[B27] Poon RT Ngan H Lo CM Liu CL Fan ST Wong J Transarterial chemoembolization for inoperable hepatocellular carcinoma and postresection intrahepatic recurrence J Surg Oncol 2000 73 2 109 114 10.1002/(SICI)1096-9098(200002)73:2<109::AID-JSO10>3.0.CO;2-J 10694648

[B28] Portolani N Coniglio A Ghidoni S Giovanelli M Benetti A Tiberio GA Giulini SM Early and late recurrence after liver resection for hepatocellular carcinoma: prognostic and therapeutic implications Ann Surg 2006 243 2 229 235 10.1097/01.sla.0000197706.21803.a1 16432356 PMC1448919

[B29] Shimada K Sakamoto Y Esaki M Kosuge T Morizane C Ikeda M Ueno H Okusaka T Arai Y Takayasu K Analysis of prognostic factors affecting survival after initial recurrence and treatment efficacy for recurrence in patients undergoing potentially curative hepatectomy for hepatocellular carcinoma Ann Surg Oncol 2007 14 8 2337 2347 10.1245/s10434-007-9415-7 17503155

